# High-Throughput Quantification of Phenotype Heterogeneity Using Statistical Features

**DOI:** 10.1155/2015/728164

**Published:** 2015-10-20

**Authors:** Ahmad Chaddad, Camel Tanougast

**Affiliations:** Laboratory of Conception, Optimization and Modelling of Systems, University of Lorraine, 7 rue Marconie, Metz, 57070 Lorraine, France

## Abstract

Statistical features are widely used in radiology for tumor heterogeneity assessment using magnetic resonance (MR) imaging technique. In this paper, feature selection based on decision tree is examined to determine the relevant subset of glioblastoma (GBM) phenotypes in the statistical domain. To discriminate between active tumor (*v*AT) and edema/invasion (*vE*) phenotype, we selected the significant features using analysis of variance (ANOVA) with *p* value < 0.01. Then, we implemented the decision tree to define the optimal subset features of phenotype classifier. Naïve Bayes (NB), support vector machine (SVM), and decision tree (DT) classifier were considered to evaluate the performance of the feature based scheme in terms of its capability to discriminate *v*AT from *vE*. Whole nine features were statistically significant to classify the *v*AT from *vE* with *p* value < 0.01. Feature selection based on decision tree showed the best performance by the comparative study using full feature set. The feature selected showed that the two features Kurtosis and Skewness achieved a highest range value of 58.33–75.00% accuracy classifier and 73.88–92.50% AUC. This study demonstrated the ability of statistical features to provide a quantitative, individualized measurement of glioblastoma patient and assess the phenotype progression.

## 1. Introduction

Glioblastoma is the most common tumor and most aggressive primary brain malignancy in adults [1]. The inability to perform complete surgical tumor resection and poor drug delivery to the brain contribute notably to the limited treatment options. Despite all efforts, the average of patient survival with GBM currently is thereabouts 14.6 months [2]. GBM consists of active tumor, peritumoral edema, and necrosis parts, as designated by the combination of T1-weighted (T1-WI) and Fluid-Attenuated Inversion Recovery (FLAIR) images in MRI. The active tumor region is described as the contrast-enhancing portion in T1-WI images and peritumoral edema is defined as the hyperintense region of FLAIR images, located outside the active area. The recent improvements in MRI technology using the integration of diffusion and perfusion weighted imaging have provided deeper insights into the pathological behavior of tumors [3, 4].

Until now, radiologists have used MR imaging for relatively gross disease detection. We hypothesize that radiomics with the availability techniques in image processing applied on the raw data derived from MRI can make radiological examinations more effective. In this way, automatic data computation could foster faster and effective readings of numerous types of images and classify them as normal or cancerous [5]. Such a system must have the ability to detect and extract the abnormal areas from their surroundings by automatic segmentation techniques such as multithresholding segmentation technique with the morphological image processing [6]. In terms of tumor heterogeneity, technical research has investigated this heterogeneity by quantifying its texture using numerous functions. For instance, feature extraction based on the gray level cooccurrence matrix (GLCM) with Haralick features is a popular technique used for texture analysis [7]. Then, GLCM computes the neighborhood correlations around pixels where the GLCM is calculated by the paired pixel in specific offset (distance) and phase (direction). Statistical (histogram) analysis has been established for the pixel intensity or the map of pixel orientations. In this context, the statistical approach used has been the texture-based approach [8–12]. Previous studies of GBM assessment have required registration of T1-WI and FLAIR for identifying the phenotypes, and each of the visible phenotypes is segmented manually by board certified radiologists.

In this paper, a novel approach for analyzing GBM phenotypes using FLAIR images only is introduced. Histogram based statistical features can offer a simple way to characterize GBM heterogeneity across the phenotypes, namely, *v*AT and *vE*. Reproducible quantifiable imaging features of GBM heterogeneity that explicitly examine links between the imaging findings and the underlying GBM phenotypes characteristics are identified. Introduced quantitative histogram features can discriminate phenotype heterogeneity from MRI images and thereby strengthen personalized medicine in GBM [12].

## 2. Materials and Methods

**Figure 1:**
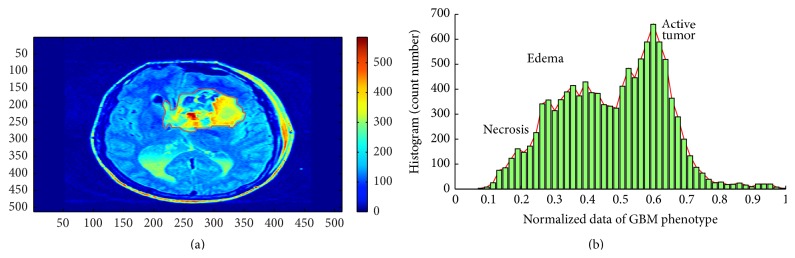
Histogram of the GBM tumor. (a) Raw image of FLAIR sequence; (b) two Gaussian distributions represent *vE* and *v*AT  and necrosis parts which are located inside the *v*AT with lower intensity values.

**Figure 2:**
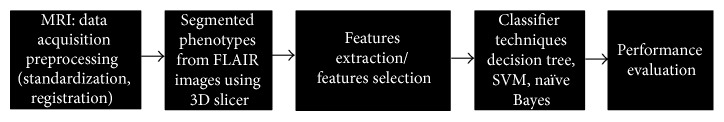
Block diagram of the proposed approach.

To prove the hypothesis, we focused on the optimal subset features from the statistical features which are derived from GBM tumors using active GBM portion with high intensity pixels *v*AT and peritumoral *vE* of GBM with middle intensity pixels. Two Gaussian distributions could clearly be observed in the histogram data of GBM (Figure 1). To assist automated recognition of the GBM phenotype based heterogeneity, histogram statistical features and classifier techniques were used for discriminating active tumor parts from edema parts in FLAIR images. Decision tree was considered to recognize the dominant statistical features which represented the foremost characteristic of GBM heterogeneity [13]. The proposed approach is presented in Figure 2.

### 2.1. Patient Information, Data Acquisition, and Segmentation

**Figure 3:**
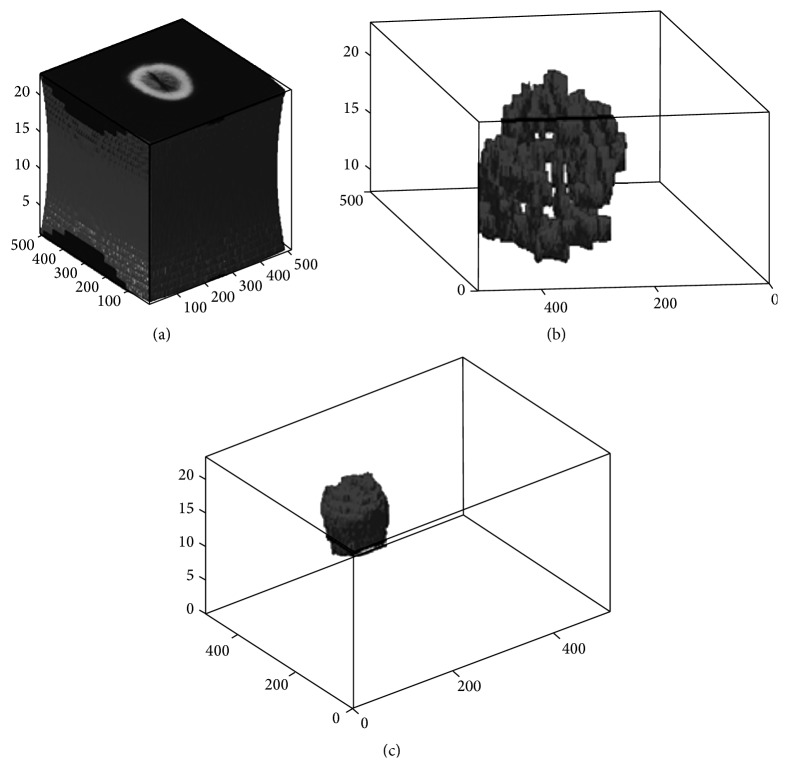
Example of phenotype segmentation. (a) Raw image of FLAIR sequence, (b) edema part *vE*, and (c) active tumor *v*AT.

After excluding samples with incomplete data, a set of 30 patients was randomly selected from The Cancer Imaging Archive (TCIA, http://www.cancerimagingarchive.net/) publicly available database for a preliminary study. To obtain full imaging sets, 30 other GBM patients' data (age 50–68 years; 15 males, 15 females) were additionally chosen randomly from the TCIA database. Image pixels of the tumor regions were independently normalized on a scale from 0 to 1 (e.g., Figure 1(b) where the* x*-axis represents the normalized tumor pixels). The images were transformed into gray scale (using Matlab 2013 software) before further processing. Only FLAIR sequences were considered in this study. All the images were reconstructed to 512 × 512 matrices by segmenting the appropriate area of GBM by board certified radiologists, using the 3D slicer tool (Figure 3) [14]. Moreover, *vE* and *v*AT phenotypes were segmented manually slice-by-slice and organized in order to extract the statistical features. Statistical features of edema and active tumor parts were extracted then from raw FLAIR images.

### 2.2. Statistical Features Extraction

Features were extracted from the histogram shape, which is an area of the variable description based on the shape, and provided the frequency of values from different ranges of the variable. These features were applied previously in cervical cancer diagnosis using histogram based analyses of diffusion-weighted MR and its relation to histological features, subtype, and grade of cervical cancer [15, 16]. We quantified the two GBM phenotypes by nine statistical functions (Table 1).

All GBM patient data were plotted as histograms showing individual GBM data and their respective frequencies (Figure 1(b)). Features describing major statistical characteristics of these distributions were extracted according to Table 1 [15]. All features were extracted from histograms of GBM, according to

(1) $$\begin{array}{c} R_{vAT}=\left[\begin{array}{ccc} f_{1,1} & \cdots & f_{1,9}\\ \vdots & \ddots & \vdots \\ f_{m,1} & \cdots & f_{m,9} \end{array}\right], \end{array}$$

where *m* is the number of *v*AT samples, for each patient's one feature vector included nine features:

(2) $$\begin{array}{c} R_{vE}=\left[\begin{array}{ccc} f_{1,1} & \cdots & f_{1,9}\\ \vdots & \ddots & \vdots \\ f_{k,1} & \cdots & f_{k,9} \end{array}\right], \end{array}$$

where *k* is the number of *vE*, for each sample, and is similar in size to the nine features.

Note that the feature value represents the average of corresponding values of all slices in each patient.

One matrix vector *R* is organized according to

(3) $$\begin{array}{c} R=\left\{\;R_{vAT},R_{vE}\;\right\}. \end{array}$$

For the GBM heterogeneity analysis, the aforementioned histogram features were extracted from the FLAIR MR images corresponding to the heterogeneity of *v*AT and *vE*. Therefore, the length of the resulting feature vector was nine. This statistical feature vector was taken as GBM heterogeneity based on *v*AT and *vE*, for the classification task at hand.

### 2.3. Statistical Analysis

Features were normalized using *Z*-scores which convert each of the feature vectors to have zero mean and unit variance. Moreover, an ANOVA test was used to assess the statistical significance between features and phenotypes [17]. This test was used to select the feature where *p* value < 0.01 was considered significant. Note that the total statistical features were found to be significant which are reported in Table 2.

### 2.4. Classifier Setting and Performance Metrics

Supervised technique such as the support vector machine (SVM) [18], naïve Bayes (NB) [19], and decision trees (DT) classifier [20] has become a popular learning algorithm for data mining applications, as employed to classify *v*AT from *vE*. A leave-one-out cross-validation was applied to obtain closely unbiased estimates of classification error rates. Additionally, receiver operating characteristics (ROC) curves and the corresponding areas under the ROC curve (AUC, with a cut-off value of 0.5), classifier accuracy, and confusion matrix were calculated to determine the performance of statistical feature for predicting the two GBM phenotypes.

*Classifier accuracy* measures the new sample correctly classified. It can be determined by the following expression:

(4) Classifier  accuracy=vAT  and  vE  samples  correctly  classifiedTotal  number  of  samples=TP+TNTP+FP+TN+FN,

where the true positive (TP) and the true negative (TN) are the number of *v*AT and *vE* samples correctly classified into positive and negative classes. The false positive (FP) and false negative (FN) are those samples which are incorrectly classified. Then, TP + FN are the total number of test samples of the considered class.

The results of the performance metrics reflect the value of this study in which the histogram (statistical) based features could be promising in discriminating between both types of GBM heterogeneity (*v*AT and *vE*). Due to this limited accuracy based on a full feature set, DT to find the optimal subset features was considered in order to improve the classifier accuracy. Simulation results were reported in Tables 3 and 4.

### 2.5. Features Selection Based on Decision Tree

Dominant features can be obtained using the decision structure “tree” model based on the general minimizing error. This model was proposed from various inducers, some comprising two conceptual phases “growing” and “pruning” (C4.5 [21] and CART [13]). The most important aspect of a decision tree induction strategy is the split criteria, which is the method of selecting an attribute that determines the distribution of training objects into subsets upon which subtrees are consequently built. The dominant features can be determined when subtrees are constructed. The choice for best attribute splitting was based on several techniques. This study used the Gini index (*I*_*G*(*t*)_) as a more effective technique for splitting data and to detect the optimal subset features. *I*_*G*(*t*)_ is an impurity-based criterion that measures the divergences between the probability distributions of the attribute's values. It is expressed as

(5) $$\begin{array}{c} I_{G\left(t\right)}=\underset{i}{\sum }p_{i}\left(\;1-p_{i}\;\right), \end{array}$$

where *p*_*i*_ is the relative frequency of class *i* at node *t* and node *t* represents any node at which a given split is performed. *p*_*i*_ is determined by dividing the total number of observations in the class by the total number of observations.

DT was applied on the given dataset using a built-in* Matlab* function from the* decision trees for regression and classification* toolbox. Comparative results were reported in Tables 3 and 4.

## 3. Experimental Results

GBM phenotypes (*v*AT and* vE*) were segmented by board certified radiologists using a manual technique of 3D slicer tools. 30 *v*AT and 30* vE* areas from raw MRI data of 30 patients were analyzed.

### 3.1. Feature Analysis

An ANOVA test showed that the nine statistical features were significant with *p* value < 0.01 (Table 2). Then, except for features *f*_4_, *f*_5_, and *f*_8_, features *f*_1_, *f*_2_, *f*_3_, *f*_6_, *f*_7_, and *f*_9_in *v*AT were significantly higher than the corresponding* vE* features. This is indicating that *v*AT is more pronounced statistically than* vE*.

### 3.2. Feature Selection

**Figure 4:**
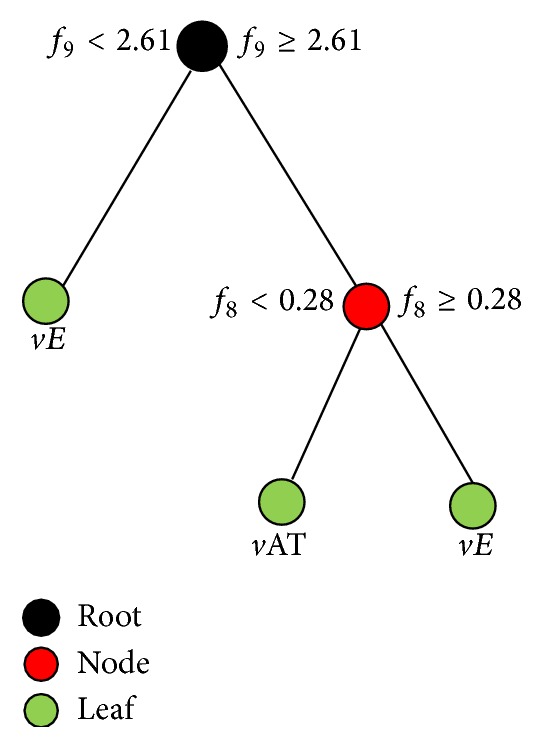
Decision tree grown using 9 statistical features extracted from 30 *v*AT and 30 *vE* parts.

Feature selection was performed by decision tree model to determine the dominant statistical features, which would provide reliable discrimination between *v*AT and *vE*.  Figure 4 shows the resulting decision tree. We observed that Kurtosis (*f*_9_) and Skewness (*f*_8_) play a dominant role as they appear towards the top of the tree structure. However, the other features have been identified as irrelevant attributes for the classification problem at hand since they do not appear in the tree.

### 3.3. Classification and Performance Comparison

A comparative study was done using the three-classifier model based on full feature set and subset feature.  Table 3 shows 53.33–68.33% range of accuracy classification using full features set and 58.33–75.00% for the subset feature with a highest value achieved using a decision tree classifier. Note that the SVM and NB classifier were not promising for discrimination between *v*AT and *vE*.

**Figure 5:**
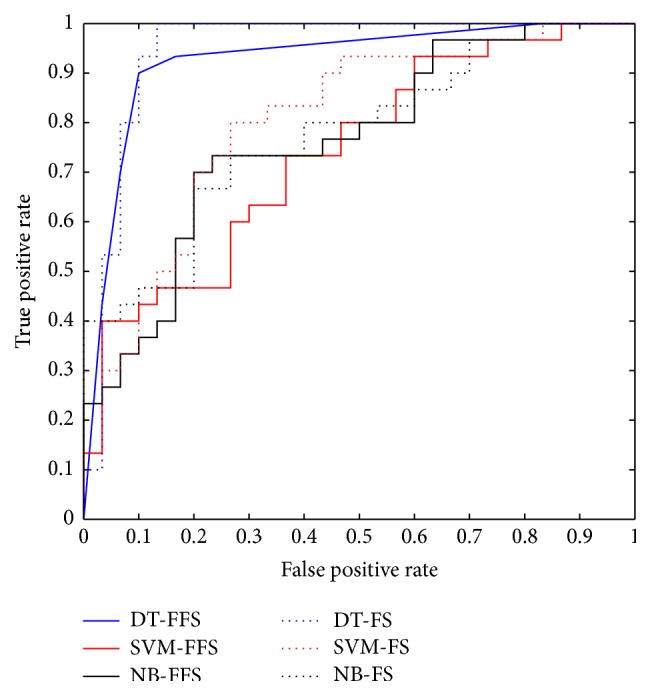
Receiver operating characteristic curves for distinguishing between *v*AT and *vE*. FFS denotes full feature set, and FS is the feature selection.

Moreover, AUC value shows a range of 77.66–96.05% for full feature set and 73.88–92.50% for subset feature with a highest value achieved using the decision tree classifier (Figure 5). This demonstrates the feasibility to discriminate between *v*AT and *vE* using the feature selection extracted from the FLAIR sequence.

Confusion matrix of the phenotypes discrimination showed that the 20 *v*AT and 25 *vE* samples are correctly classified from 30 phenotype samples based on DT classifier (Table 4). High rate of misclassification samples was in *v*AT samples where 10 of 30 samples were incorrectly classified as *vE* phenotype.

## 4. Discussion

**Figure 6:**
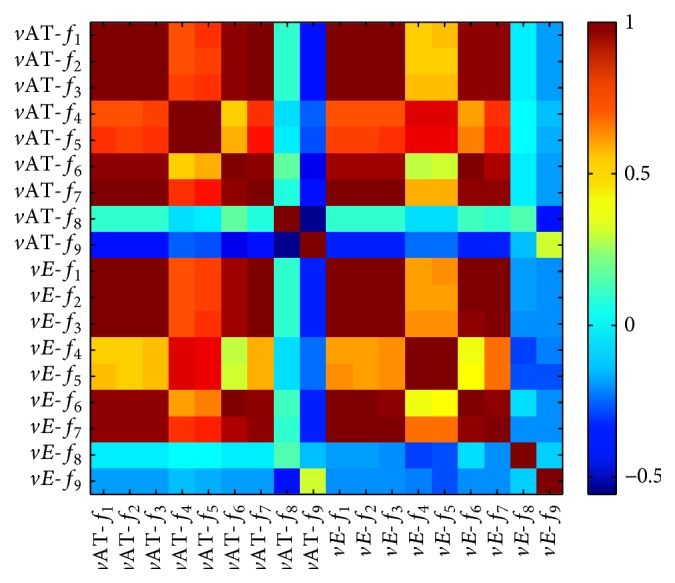
Heat map with correlation coefficients between statistical features.

In this study, we used statistical features extraction to assess *v*AT and *vE* phenotypes based on FLAIR sequence. The classifier accuracy of 75.00% was achieved using 30 patients and two features selected based on decision tree model. Among the nine features, Kurtosis (*f*_9_) and Skewness (*f*_8_) values might reflect the appropriate features which represented less correlation with other features. In this context, Figure 6 shows the heat map of the correlation coefficients between the nine features. We observed that the lowest correlation coefficients were achieved by two features Kurtosis (*f*_9_) and Skewness (*f*_8_). Note that the higher correlation coefficients represent the common characters between *vE* and *v*AT, as shown in the statistical features (*f*_1_, *f*_2_, *f*_3_, *f*_4_, *f*_5_, *f*_6_, and *f*_7_). These two selected features can be associated with the phenotype heterogeneity.

In this context, multiple studies have suggested that increasing heterogeneity is associated with cancer [22]. The results demonstrated that building features and biological significance are promising for noninvasive detection of GBM heterogeneity based on *v*AT and *vE*.

Moreover, recent efforts concluded that future research will be most productive by focusing on genetics, clinical data, and imaging features [23]. Thus, characterizing the molecular properties of GBM and making them publicly available are goals of the Cancer Genome Atlas (TCGA) [24].

Traditionally, the feature extraction applied to medical imaging was limited to the whole tumor and normal brain [23]; however, current advances in medical image processing like the findings presented in this study allow for high-throughput extraction of characteristic imaging features to measure complex and very subtle differences across patient MRIs. Thus, these findings provide strong evidence that feature extraction can identify and discriminate between GBM heterogeneity types.

Previous study was done to apply texture analysis for assessment of the traumatic brain injury [25] and also to discriminate between the GBM phenotypes; however, two MRI sequences were used, features of necrosis and active tumor parts from T1-WI images and edema parts from FLAIR sequence. The texture feature extracted from GLCM was considered, and the simulation results for 13 patients show the highest accuracy of 67% [26].

Obviously, neuroradiologists are becoming increasingly important players for early diagnosis of GBM. Our vision is to integrate engineering based methods as described in daily practice to enhance radiologists' performance beyond their routine “vision.” Particularly, in utterly devastating disease like GBM, improvements in any medical specialty involved are of the utmost essence. Note that numerous factors may have led to varying results between this study and previously published studies potentially due to the following reasons.

In this work, the whole GBM tumor was assessed. Only FLAIR sequence was considered and only two phenotypes were addressed based on the data distribution (Figure 1). It would have been preferable to include more patients to strengthen the GBM heterogeneity analysis. However, the number of patients included in this study can provide preliminary information about GBM heterogeneity.

## 5. Conclusions

This paper analyzed and implemented GBM *v*AT and *vE* discrimination based on the statistical features extracted from MRI raw data. For the analysis of GBM heterogeneity, feature extraction was more effective as it could characterize each phenotype by a specific set of features to robustly identify them. By automatic recognition, this identification subsequently provided a more accurate assessment of the patient prognosis and underlying genomic composition. Improved classifier accuracy was achieved using the decision tree model. Feature extraction, selection, learning, and classification were applied on 30 *v*AT and 30 *vE* phenotypes.

The experimental results were confirmed by higher accuracy classifier of appropriate features based on two features Kurtosis and Skewness. The drop in average correct classification rate resulted from difficulty in classifying based on histogram and subset feature. Histogram feature extracted from GBM phenotypes yields a promising technique for differentiating *v*AT from *vE* indicating the oncological level of aggressiveness of a tumor. Extending this work by increasing the number of patients would enhance the accuracy of GBM heterogeneity prediction in the future.

## Figures and Tables

**Table 1:** Statistical features description.

Symbol	Features
*f*_1_	Geometric mean, indicates the central tendency

*f*_2_	Harmonic mean, calculates the average sample

*f*_3_	Mean excluding outliers, measures the probability distribution

*f*_4_	Mean (average)

*f*_5_	Standard deviation (absolute deviation)

*f*_6_	75th percentile, splits off the highest 25% of pixels from the lowest 75%

*f*_7_	Quantile

*f*_8_	Skewness, assesses the asymmetry of the distribution

*f*_9_	Kurtosis, measures the degree of peakedness of a distribution

Features vector	*F* = {*f*_1_, *f*_2_, *f*_3_, *f*_4_, *f*_5_, *f*_6_, *f*_7_, *f*_8_, *f*_9_}

**Table 2:** Mean ± standard deviation of *v*AT and *vE*.

Features	*v*AT	*vE*	*p* value
*f*_1_	473.02 ± 345.65	461.15 ± 341.98	<0.01
*f*_2_	466.49 ± 344.81	453.57 ± 342.15	<0.01
*f*_3_	478.88 ± 347.18	468.25 ± 342.53	<0.01
*f*_4_	47.50 ± 31.33	53.69 ± 31.15	<0.01
*f*_5_	37.92 ± 25.98	44.19 ± 25.18	<0.01
*f*_6_	327.31 ± 294.16	321.74 ± 302.46	<0.01
*f*_7_	519.27 ± 367.27	516.73 ± 359.66	<0.01
*f*_8_	−0.20 ± 0.29	−0.09 ± 0.40	<0.01
*f*_9_	3.58 ± 0.85	2.93 ± 0.59	<0.01

**Table 3:** Metrics (%) of *v*AT and *vE* discrimination.

Features	DT		SVM		NB	
	Accuracy	AUC	Accuracy	AUC	Accuracy	AUC
Full feature set (*F*)	68.33	96.05	68.33	80.22	53.33	77.66

Subset feature	75	92.5	58.33	73.88	58.33	76.44

**Table 4:** Confusion matrix based on selected features.

Features	DT		SVM		NB	
	*v*AT	*vE*	*v*AT	*vE*	*v*AT	*vE*
30 *v*AT	20	10	15	15	13	17
30 *vE*	5	25	10	20	8	22
